# Evaluation of a new lactation device ‘Lactamo’ designed to apply massage, heat or cold, and compression to the breast

**DOI:** 10.1186/s13006-022-00466-9

**Published:** 2022-03-24

**Authors:** Linda Sweet, Vidanka Vasilevski

**Affiliations:** 1 School of Nursing and Midwifery, Deakin University, 221 Burwood Highway, Burwood, Victoria 3125 Australia; 2 Centre for Quality and Patient Safety Research, Western Health Partnership, 176 Furlong Road, St Albans, Victoria 3021 Australia

**Keywords:** Breastfeed, Breastfeeding, Massage, Heat therapy, Cold therapy, Compression, Lactation

## Abstract

**Background:**

Common approaches to manage breastfeeding problems such as pain, blocked ducts, and milk production issues include breast compression, breast massage, application of warmth or cold, medications, and breastmilk expression. Several devices are available to apply heat or cold to the breast, however, none promote breast compression and/or massage simultaneously. A new device ‘Lactamo’ has been developed to address this.

**Methods:**

This study was a pre-market evaluation of the Lactamo device. The aims were to determine user safety, and satisfaction of Lactamo. The study was conducted in an Australian tertiary maternity hospital in 2019–2020. Women who were less than 3 months post-partum and were currently breastfeeding participated in the study. We conducted structured telephone surveys at 1 and 4 weeks post supply of Lactamo. Questions included demographic information, feedback on safety, usage, and perceived benefits of Lactamo.

**Results:**

The cohort (*n* = 30) consisted of equal number of primiparous and multiparous women, 50% were born in Australia and the remainder from 11 other countries. A total of 41 telephone surveys were conducted with 27 women. Of these, 26 (96%) had used Lactamo, and the one that did not, felt she did not have a lactation concern to warrant using it. All women indicated that the device was safe to use and had no concerns, apart from one woman who experienced itching because of the device but continued to use it over clothing as she found it beneficial. Most women used it at room temperature or warmed. The frequency of use varied from once per week (17%) to daily (33%), and use was often prompted by a lactation concern such as engorgement, pain, blocked ducts, and low supply.

**Conclusion:**

Lactamo was found to be safe, and a valuable aid for breastfeeding women. More research is needed to understand the efficacy of the device in treating breastfeeding problems such as pain, blocked ducts, and milk production issues.

**Supplementary Information:**

The online version contains supplementary material available at 10.1186/s13006-022-00466-9.

## Background

Nearly 90% of Australian mothers initiate breastfeeding at birth or soon after [1]. However, within weeks or months many mothers stop breastfeeding. The reasons women cease breastfeeding are numerous and multifactorial and can often be overcome with effective treatment and support. Breastfeeding problems without effective treatment, such as blocked ducts, can lead to mastitis, breast abscess, and ultimately breastfeeding cessation [2]. Mastitis is just one example of an acute debilitating condition that occurs in approximately 17% of breastfeeding women [3]. Common approaches to managing breastfeeding problems such as blocked ducts, engorgement, pain, mastitis and milk production issues include breast compression, breast massage, application of warmth or cold, use of medications, and breastmilk expression [3–7]. A recent systematic review of breast massage for the treatment of women with breastfeeding problems reported a reduction in pain regardless of the breast massage technique used [8]. Overall, varied types of breast massage were helpful in reducing immediate pain and resolving symptoms of blocked ducts, engorgement, and mastitis [8]. The included studies used unstructured or structured hand massage interventions, with or without heat packs, or massage combined with a massage medium such as crushed aloe and cactus [8]. The review included no studies using a device to assist in breast massage.

Application of warm and/or cold packs are other interventions commonly used in the management of conditions such as engorgement, blocked ducts, and mastitis independently or in conjunction with massage and/or expression [9–11]. One study compared cold cabbage leaves and cold gel packs, and found both relieved pain and hardness in breast engorgement, with the former having a better effect [10]. Similarly, symptoms of breast engorgement improved with the application of warm and cold compresses, in conjunction with frequent breast emptying and breast massage in another study [9]. It is common advice to apply heat therapy before or during a feed/expression to encourage milk flow and release blockages for engorged or swollen breasts, and cold therapy after a feed/expression to provide pain relief or to reduce inflammation or swelling [7]. Current lactation support devices on the market are primarily disk-shaped gel packs that can be applied to the surface of the breast or be rested inside a bra. None of these are designed as a concomitant massage device.

**Fig. 1:**
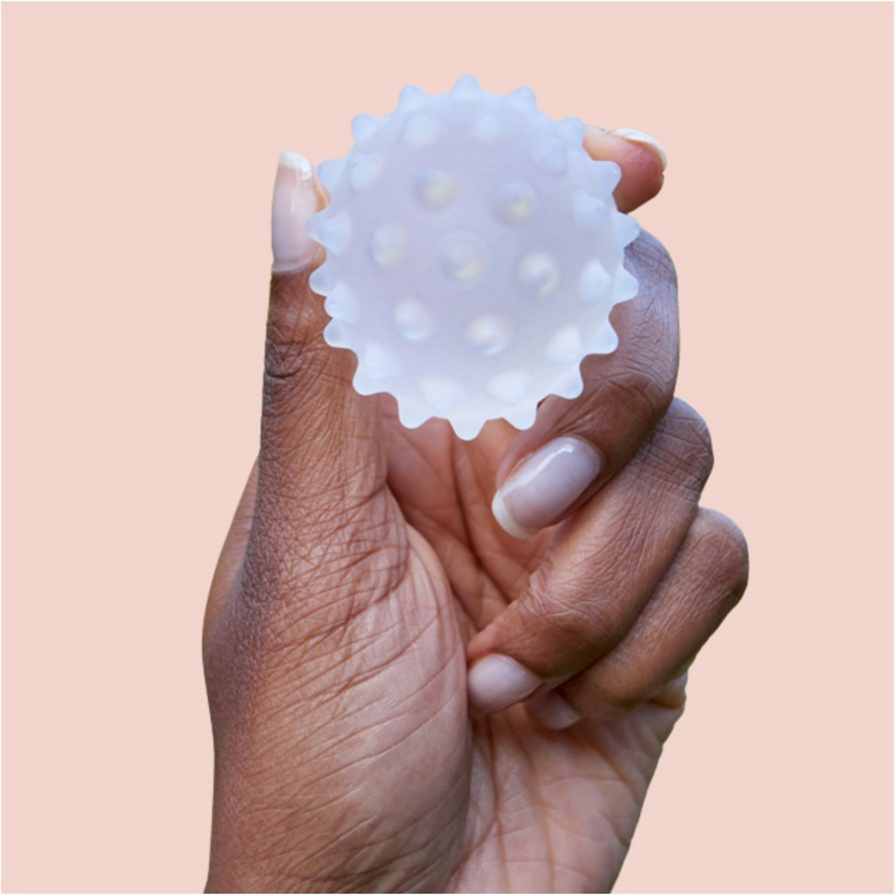
The Lactamo device

Lactamo is a new ‘lactation aid’ which was designed to be a therapeutic device for women with breastfeeding problems. It combines massage, warm/cold therapy, and compression. Lactamo’s inventor is a mother of four children with first-hand practical and emotional experience of challenges that many breastfeeding mothers face. Lactamo’s name is derived from the Latin words ‘lact’ meaning milk, and ‘amo’ meaning friend. It has been designed to address common breastfeeding problems reactively and proactively. Lactamo is a small (5 cm) soft sphere filled with thermochromic hot/cold gel and hollow surface protrusions (see Fig. 1). The protrusions are of varied firmness on each side of the device, allowing the user to decide their own level of compression pressure. It can be warmed in hot water or a conventional steam steriliser or cooled in a fridge or freezer. Applied directly to the breast by the user, Lactamo allows the combination of three critical components—temperature, movement, and compression—which can be applied and adapted as and when desired. It is used by rolling over or rotating on the breast tissue in movements toward the nipple, axilla, or in a specific problem area.

Lactamo, to our knowledge, is the first device of its kind, facilitating breast massage with the capacity to apply both variable pressure and desired temperature at the same time. Traditional approaches such as massage and applying heat or cold at the breast are considered beneficial strategies for managing common breastfeeding problems [5, 8, 10–16]. There are a range of devices on the market for the purpose of warm/cold therapy. Examples include Lansinoh Therapearl, Mumasil Reusable Warm and Cool Breast Packs, Rita Aid Hydrogel Breast discs, and BodyICE Woman. The instructions for use of these products commonly include the application of heat therapy for engorgement or swollen breasts immediately before feeding, and cold therapy between feeds to help reduce swelling and provide relief. For blocked ducts or mastitis, they recommend applying heat therapy and gentle massage to the affected area between and during feeds, and cold therapy between feeds to help reduce pain and swelling. All these devices are disk shaped gel packs that can be applied to the surface of the breast or be rested inside a bra. None of these are designed as a concomitant massage device. A device that facilitates these approaches simultaneously, such as Lactamo, may be useful for supporting women to overcome common breastfeeding concerns such as engorgement, pain, blocked ducts, and supply issues.

This pre-market evaluation aimed to investigate the safety, use, and satisfaction of Lactamo.

## Methods

### Study aims

The primary aim was to evaluate the safety of the Lactamo device. The secondary aims were to determine:

- the percentage of women who used the Lactamo device
- women’s satisfaction with using the Lactamo device
- how often and how long women used the Lactamo device
- the range of lactation concerns for which women used the Lactamo device

### Study design

An exploratory mixed-methods approach was used. This approach is suitable for pre-market evaluation of an unapproved therapeutic good (medical device) [17].

### Study setting

Women were recruited from a large tertiary maternity hospital in Melbourne, Australia between November 2019, and January 2020. The hospital is in a culturally diverse and socioeconomically disadvantaged area, where encouraging the maintenance of breastfeeding is an ongoing challenge [1].

### Participants

Inclusion criteria required participants to be women over 18 years of age, able to speak conversational English, had given birth within the last 3 months, were breastfeeding or expressing breastmilk with the intention to continue for at least 4 weeks. A convenience sampling approach was used, whereby women were identified from the daily inpatient lists and those meeting the criteria were invited to participate in the study by one of the hospital lactation consultants or a member of the research team. Women from both the postnatal wards and the neonatal services were approached for the study to promote variation in the sample. Women were given details about the study and were shown the Lactamo device and how it should be used. Those who were interested in being involved were provided with a participant information sheet and a consent form for their consideration. Following informed consent, participants were provided with a Lactamo device, along with a research information flyer and instructional materials. Their name and preferred contact telephone number were included on the consent form for the researchers to contact them for the study. Recruitment ceased when the sample size of 30 was reached. This sample size was selected to follow the Australian clinical trial guidelines for medical devices which recommends 10–30 participants in a pre-market pilot study (p44) [17].

### Data collection

A structured questionnaire with 24 questions was developed to elicit the information required to address the aims and outcome measures. As data was collected via telephone. Prompts were used to obtain depth of understanding based on the participants response. For example, when asked “did you use Lactamo” if a yes response was given a follow-up question such as “in what ways” was asked. Data were collected at two time points: approximately 1 week (T1) and 4 weeks (T2) post supply of the Lactamo device. At T1, 20 of the 24 questions were asked, and at T2 all 24 questions were asked. Questions included demographic information, past-experience with breastfeeding (for multiparous women), presence of any current lactation concerns, current infant feeding practices, woman’s experiences of the safety of the Lactamo device, when, where and at what temperature Lactamo was used, and feedback about the Lactamo device and the instructions. Infant feeding practices were characterised as exclusive breastfeeding, breastfeeding and expressing, expressing only, formula feeding, and breastfeeding with formula supplementation. At T2 additional questions included perceptions of the device design, recommendations for improvement, and whether they would recommend the product to other mothers. Data were collected by the principal researcher, a former lactation consultant and midwife. As the women were surveyed over the phone, the interviewer input data into the online survey tool during the conversations where possible. The conversations were also recorded to enable data collection to be checked for accuracy. Each interview was re-listened to, and the data entry edited as necessary to ensure accuracy, with a sample being double checked by author 2. During the analysis phase the researcher used both the text data and re-listened to the recordings to ensure context and accuracy.

### Data analysis

Descriptive statistics were used to analyse quantitative data. Categorical variables were presented as frequency and percentage. Continuous variables were demonstrated using median and range values. Content analysis was used for the qualitative data. Content analysis is a systematic and objective means of describing and quantifying qualitative data [18, 19]. An inductive approach including open coding, to create categories and abstraction was undertaken using NVivo 12 software [18]. Initial codes and categories were reviewed and discussed by both researchers until agreement was achieved. Where quotes are provided in the results section, each participant is identified by their participant number (i.e., P1, P2). Given the pre-market evaluation design, data saturation was not relevant.

### Ethics approval

Ethical approval (No. 2020.259) was gained from the high risk Melbourne Health Human Research Ethics Committee as the device had not yet been listed on the Australian register of Therapeutic Goods. Reciprocal approval was provided through Deakin University (2020–367) and local site governance approval (No. HREC68195MH2020) at the study setting. As this was a pre-market evaluation of a Class 1 external device, trial registration was not required. Participation was voluntary, all women provided informed consent, and were able to keep the Lactamo device at the conclusion of the study.

## Results

Thirty women were recruited into the study. Of these, three were lost to follow-up and 13 women participated in only one of the two scheduled interviews. As a pre-market pilot we did not replace those lost to follow-up, so the final sample included 27 women. There were 22 interviews at T1, and 19 at T2. The characteristics of the 30 recruited participants recruited are shown in Table 1 and in the additional file available online. The women’s ages ranged between 27 and 40 years with a median of 33 years. For half the women, this was their first baby. Most women gave birth at term, 30% had a preterm birth.

**Table 1:** Participant characteristics (*N* = 30)

Characteristic	Variable	N (%)	Median (Range)
Age (years)			33 (27–40)
Parity	Primipara	15 (50)	
	Multipara	15 (50)	
Maternal country of birth	Australia	15 (50)	
	Other^a^	15 (50)	
Mode of birth	Vaginal	15 (50)	
	Caesarean Section	15 (50)	
Gestation at birth (weeks)			38 (28–41)
	Preterm (< 37)	9 (30)	
	Term (+) (> 37)	21 (70)	
Previous breastfeeding experience	Yes	16 (53)	
	No	14 (47)	
Recruit time (days post birth)			3 (0–69)
	Within 7 days	24 (80)	
	More than 7 daysb	6 (20)	

^a^Other included: three from India, two each from New Zealand and Vietnam, and one each from China, Egypt, Hong Kong, Malaysia, Nepal, Pakistan, Samoa, and Singapore

^b^5 of the 6 gave birth to preterm infants and one was a term infant, all infants were in the neonatal unit

### Reasons for use

The reasons women chose to use Lactamo varied, but for most participants who used it (*n* = 25/27; 93%), a lactation condition or situation prompted its use (see Table 2). Most women (*n* = 24/27; 89%) experienced more than one lactation concern, and 17 of 27 (63%) women had at some time expressed their breastmilk. Fourteen of 27 women sought advice or reassurance from a health professional at some point in their lactation journey, and one woman spoke of how “excited” her midwife was about the device (P3). No health professional discouraged the use of Lactamo.

**Table 2:** Frequency of conditions/situations that prompted the use of Lactamo (*N* = 26)

Condition/situation	Yes N (%)
Engorgement/full breasts	17 (65)
Blocked ducts	13 (50)
Breast pain	11 (42)
Promote let down	7 (27)
Breast emptying	5 (19)
Promote milk production	5 (19)
Mastitis	3 (12)
Breast abscess	1 (4)
Nil	2 (8)

The most common lactation concern that prompted the use of Lactamo was the sense of full and painful breasts or engorgement. All the women experiencing these concerns found using the device very helpful. One mother said, “*when my breasts are engorged, I think it definitely helps get the milk out” (P4).* Similarly, it was seen as useful when experiencing blocked ducts and pain. One mother explained, *“I think it helped the milk come out easier and helped the breast pain. I used the ball to massage out the lumps and it worked” (P11).* For some it was used as a preventative tool.

*“I have used it more in the last couple of weeks because I had a bit of pain from time to time on both sides, so I wasn't sure if I had a blockage, so for me I use it as a preventative and a method of relieving pressure or pain” (P2).*

Some women chose to use it prior to feeding or expressing to promote let down and breast emptying. As explained, *“I tried it while feeding … and while pumping. It was better using the ball [Lactamo] and it was warmer and released the milk more easily [than hand massage]” (P10).* One woman found using Lactamo shortened her expression time. She explained, *“I massage with it – [I get the] same volume but in less time pumping” (P17).* The two women who used it with no lactation concerns, tried it *“just once or twice” (P12)*, or used it while expressing (P21) a few times but did not continue to use it as felt they did not need it.

### Usage, safety, and comfort

Of the 27 participants, all but one had used Lactamo (*n* = 26/27; 96% usage rate). The one woman who did not use it, explained *“I did hold it and try rubbing on the breast, but not properly, I had no reason for using it, … I am ok without it”* (P5).

There were no safety concerns raised by any of the participants, although one tolerance concern was raised. One woman found that when she applied the device directly to her skin, she became “very itchy”. However, she found the device beneficial for softening her breasts before expressing and continued to use it over her bra. She explained,

*“I don’t use it directly on my skin. I have very sensitive skin and the few times I tried directly on the skin my breasts became very itchy – it’s not like I am allergic to silicon or anything, I think it is the spikes, you know, … too spikey for me against the bare skin. But I use over the bra and that minimises the problem - I don’t find myself itchy after that. I don’t think it is an issue about contact with the material” (P17).*

All the participants (*n* = 27/27) felt the device to be comfortable to use on their breasts. Most women described Lactamo as a soft and soothing device.

*“Very comfortable because it’s really soft. Other massage balls are a bit firm - this one is so easy to roll on the breast even if I push harder - with other massagers its painful – with this one it’s just easy” (P19).*

Lactamo is designed with small protrusions over its surface, with one side being firmer than the other. Apart from the one woman who was intolerant of the protrusions on her skin, all other women found the protrusions comfortable and a benefit of the device. One woman said, “t*he protrusions don’t bother me, I think they help with the massage. I haven’t had an issue with the spikes at all” (P20),* and another, *“I like that the spikes vary on each side - a softer side and a spikier side” (P2).*

A few women spoke of non-lactation uses for the device. Some women spoke of its relaxation benefits, as explained, *“it’s like an all in one device - stress ball for me and a relief especially when my breasts were full … I hold it all the time, especially when tired” (P19).* One mother said, *“it can be used for baby massage also” (P4).*

### Frequency and timing of use

The frequency of use varied considerably from once per week (*n* = 6/27, 23%) to daily (*n* = 9/27, 35%). Of the nine women who used it daily for a period of time, seven women (recruited in the first week postpartum) used it daily in the first one to 2 weeks of their lactation and then reduced frequency, one woman (recruited at 31 days postpartum) used it daily as she perceived it to improve milk production while expressing, and another woman (recruited at 69 days postpartum) used it daily to relieve symptoms of blocked ducts, mastitis, and a breast abscess. For all women, once their lactation concern had been relieved, they reduced their use of the device.

*“I don’t use it every time nor every day. I use it when I missed my timing for pumping and my breasts get engorged and hard, and you can feel the ducts, so I use the ball to soften it up. I think it does a really good job - so it is in my pumping kit all of the time” (P17).*

Lactamo was mostly commonly used in the early weeks of establishing lactation.

*“It definitely helps, particularly in first few weeks when I was still finding my mojo - at that point, I have never had issues with supply, but lots of lumps in my breasts, so it was really useful to help get those out. I was quite engorged and in pain” (P13).*

Of those who used Lactamo, most chose to use the device before feeding or expressing (*n* = 18/26; 69%), or during a feed or while expressing (*n* = 16/26; 62%), while 42% (*n* = 11/26) said they had used it after a feed. One mother used it before a feed *“to get things softer and to help put baby on” (P14)*, but when trying to use during a feed she found *“I just tend to drop it and it goes in between things and the baby is on me and I can’t move” (P14).* Some women (*n* = 6/26, 23%) described using it in the shower, as one woman said,

*“I use in the shower as I find it more convenient, and easy to add to the routine. Massage to improve supply, the warmth of water helps too - it just feels a lot better, my comfort with it [Lactamo] depends on pressure and fullness of the breast” (P25).*

Of the 11 women who used Lactamo after a feed (*n* = 11/26; 42%), ten of them had said they used it for lactation concerns including engorgement, blocked ducts, mastitis, and/or pain, while three used it to promote supply. It is unclear whether these women used the device warm or cold when using after a feed, and which of these concerns prompted its use.

Most of the women said the device did not interfere when used at the time of their breastfeeding or expressing. One mother explained how she ensured good attachment before commencing to use the device during feeding, she said *“No, I don’t think it interrupted, after he latches, I then start using the ball to massage while he is feeding” (P26).* One woman at times required help to apply it and another thought help would be beneficial. She said, *“my partner would help me use it during a feed, so I had my hands free” (P18)*, and another said *“I found it awkward - maybe I was doing it wrong. If I had a third hand it would be so much easier!” (P13).*

### Temperature of use

Of the women who used Lactamo, 19 did so at room temperature, 11 used it warmed, and 3 using it cooled. Heating the device was easy for most, one woman explained, *“I tried heating it under the tap but prefer microwave as it stays warm for quite a while. Temperature stays even when warmed in microwave” (P3)*. Most women used a microwave or hot water to heat the device, although one mother commented, *“I am scared to put in the microwave” (P12).* The practicalities of heating the device were noted, with one woman saying, *“if I am bothered, I like to heat it in the microwave or hot water, if not, I just use it at room temperature” (P3)*. Similarly, P6 said *“I use it at room temperature because there just wasn’t time”*. A few women recognised the therapeutic benefits of using the device warm, such as *“I like it warmed to help get the milk down” (P10).* The women who tried using the device cold did not explain why – just that they wanted to *“try everything” (P4)*.

### Perceived benefits

The women (*n* = 26/27; 96%) who used the device felt that it was beneficial for their lactation. One woman said,

*“Yes – it was actually really good, and I had the mastitis, and it actually did help really well. … With the milk supply, perhaps it’s helping with the milk movement as well as I am having more supply. So, when I was getting mastitis. The milk was really slowing down but it is building up again. … I guess it helped, the massage ball [Lactamo] was helping break down the fluid from the abscess. I am still actually using it right now so when I breastfeed, I massage it around and it’s really relaxing by itself … it’s really fantastic!” (P19)*

Some women saw Lactamo as an adjunct to other measures such as hand massage, *“I feel I can get into area [with Lactamo] when hand massage can’t, and it is a different technique, like a secondary method” (P2)*. Although for most it was seen as better than hand massage, as explained, *“it’s easy to use, and it beats having to use your fingers for massage – you just roll it around – so it’s easier and more effective” (P9).*

One participant was unsure if it was of benefit but continued to use the device 2–3 days per week. She said, *“I’m not entirely sure, as I have been expressing, so I don’t know if it’s the expressing, or the lactation cookies I am taking, or if it is the ball that has helped my supply” (P25)*.

### Recommendations for improvement

During the T2 interview (*n* = 19), all participants were asked additional questions about the instructions and device, recommendations for improvement, and whether they would recommend the product to other mothers.

Most women ‘glanced at’ or read the instruction sheet when first receiving the device, however, none went back to these or used the provided QR code to review demonstration videos. It was thought that *“it’s pretty easy to use, it’s not complicated” (P26).* No recommendations for improvement of the instructions were offered, as they were deemed *“easy to follow” (P10).*

The size and texture of Lactamo were reviewed by these women. Most women felt the size and texture were good. One mother explained, *“I think the size is convenient. I like the texture in terms of how soft it is – I like [the] option to heat or cool it down if needed” (P1),* and another said, *“I think it is a good size, and I like the soft texture – it’s hard enough to work” (P9).* However, six (*n* = 6/19; 32%) suggested a little bigger size may be of benefit for some women. One woman explained,

*“I think maybe the size could be increased a little bit, or perhaps a range of sizes based on how big the mum is. For me personally, I have big boobs [breasts], if I had one a little bigger, I may have found it easier to move around” (P2).*

Six women reported it would be beneficial to have two Lactamo devices. This was so that they could either massage both breasts simultaneously, have two of different sizes, or to have one warm and one cold at the same time. Most of the mothers who thought they would use two simultaneously were expressing with a double pumping bra or used the device in the shower.

The packaging was raised by two people, as the prototype was provided in a cardboard container. One mother said, *“I find the cardboard thing really annoying - something that could be kept sterile would be more useful so when I take it out, I know it is clean” (P13)*, another thought *“a pouch would be fine” (P17).*

When asked would they recommend the device to other mothers, there was a 100% positive response (*n* = 19/19). One mother explained, *“I think it is a decent product - it works, so that’s good” (P27),* and another, *“definitely! If you get training on it and what it can be used for, I think especially new mums who don’t know what they are doing, it will definitely benefit them” (P20).*

## Discussion

This pre-market evaluation study is the first to examine the safety, use, and perceived benefits of an innovative breastfeeding support device, Lactamo.

Lactamo was deemed to be safe by all the women in the study, and no significant safety concerns were reported. The device was also considered to be acceptable, with most women using it, and willing to try it for a range of lactation purposes and concerns. All women reported that they would recommend Lactamo to other mothers to assist their lactation.

Using Lactamo for the facilitation of massage was favoured by the women, indicating that application of Lactamo could be adjusted to meet their preferences for compression pressure. Furthermore, the device was perceived to allow greater coverage of the breast than could be achieved by hand massage alone. A recent review [20] has shown that breast massage is an effective method for managing common breastfeeding problems, however, no massage devices were included. The current observational study demonstrates that Lactamo may enhance the facilitation of breast massage as it can be easily guided around the breast. In addition, the ability to modify its temperature is an added benefit for addressing concerns, however, this function was not always used by the women. Increased milk production has been demonstrated with breast expression and areola compression with an electric expression device [21] and by hand massage during breast expression [22]. Furthermore, Oketani breast massage has been shown to increase total solids, lipids, and casein concentration and gross energy of breastmilk [23]. While the women in the study indicated that their use of Lactamo may have increased their supply, the devices’ impact on breastmilk volume and composition warrants further investigation.

Frequency of Lactamo use was variable. For some women, frequency of use was determined by the occurrence of a breastfeeding concern (e.g., engorgement), where increased application occurred until the problem had dissipated. The majority of women used Lactamo at room temperature, while this was beneficial for massage and compression, using the device at the recommended temperature for specific concerns (e.g., warmth before and during feeding for blocked ducts, and cold after feeding for breast engorgement) may have more effectively managed lactation problems [11, 14, 24, 25]. Women in this study tried Lactamo before, during, and after breastfeeding/expressing often without a clear understanding of the evidence of when it should be used warmed or cooled – depending on their symptoms. Some women indicated that applying the device warmed was preferred, however, heating Lactamo was often not conducted due to lack of time or perceived inconvenience. Whilst for the purpose of this study, women were advised to warm Lactamo in a microwave or warm water, the product is now recommended to be warmed in hot water or a steam sterilizer. A few women used Lactamo cooled, more so ‘just to try it’ than for a therapeutic indication. These findings suggest the need for greater promotion of the benefits of heating or cooling Lactamo for specific breastfeeding concerns.

Most of women glanced at the instructions, but given Lactamo’s simplicity, did not feel the need to review the instructions when particular lactation concerns arose. Despite the Lactamo website [26] having clear instructions for use, no participants went to this resource, with most women stating they just did what felt right to them to manage their lactation concern. This may be problematic if the device is used in a manner contradictory to current evidence-based practice. One clear example from this study was the woman who stated she used the device to massage her breast abscess. In the presence of a breast abscess, the current preferred management is antibiotic therapy and aspiration under local anaesthetic [25]. It is important that women receive accurate and appropriate advice for the use of any therapy to manage lactation concerns. Whilst some women will seek advice for lactation problems from a health professional, many now seek advice from social media [27–29]. Research has demonstrated that women desire evidence-based and individualised advice regarding their lactation management [30], and that people-based breastfeeding services in the community are preferred [29]. Understanding the combination therapy offered by Lactamo, massage, temperature, and compression, is an important message for health professionals and consumers to maximise the benefits of its use.

### Strengths and limitations

This is the first study to evaluate the new Lactamo device. A strength of this study is the diversity of mothers in parity, ethnicity, and gestational age of baby at birth. A potential limitation is that not all women were recruited at the same time postnatally. Whilst most women were recruited in the early days postpartum, those who were recruited later were done so as attending the hospital regularly to attend their sick baby in newborn care. Having already established lactation, they still described the device beneficial to support their lactation, although wished they had received it earlier. Three women were lost to any follow up as they did not answer the phone or return voice messages, or when contacted stating they were using it but too busy to answer questions. Thirteen women participated in only one of the two planned phone interviews. The timing of conducting the study was suboptimal as it was over the Christmas holiday period. Many women who participated in only one interview stated they were just too busy with their new baby and seasonal celebrations, or they had nothing new to say or were away on holidays. It is also possible that women agreed to participate to receive a free Lactamo without intention to respond to the research questions. As this was a small pilot study at a single study site, findings may not be generalisable to the broader population of breastfeeding women. Another limitation is the reliance on women’s recall. Future work would benefit from implementing a diary to track frequency and duration of use more precisely and conducting a randomised controlled trial to determine effects on common lactation problems such as pain, engorgement, blocked ducts, and supply issues.

## Conclusion

This pre-market evaluation has determined Lactamo to be safe to use and well tolerated. The frequency and temperature with which women chose to use it varied widely suggesting women needed more information about effective use. All women would recommend the use of the device to other mothers. As this was a pilot study to determine safety, use, and satisfaction of the product, more research is needed to understand its efficacy in resolving breastfeeding problems such as pain, blocked ducts, engorgement, and supply issues.

## Supplementary Information

**Additional file 1.**

## Data Availability

The datasets generated and/or analysed during the current study are not publicly available due to lack of ethical approval for sharing but may be available from the corresponding author on reasonable request.
