# Supplementary material for: Evaluation of a new lactation device ‘Lactamo’ designed to apply massage, heat or cold, and compression to the breast
Source: Int Breastfeed J. 2022 Mar 24;17:23. doi: 10.1186/s13006-022-00466-9 (PMC8944040; doi:10.1186/s13006-022-00466-9)
Supplement: Supplementary file 1 — Additional file 1. [file 13006_2022_466_MOESM1_ESM.docx]

# Additional file: Individual participant details

| Participant | Maternal Age (years) | Parity | Mode of Birth | Gestation at birth (weeks) | Infant age at timepoint 1 | Mode of feeding at timepoint 1 | Infant age at timepoint 2 | Mode of feeding at timepoint 2 |
| --- | --- | --- | --- | --- | --- | --- | --- | --- |
| 001 | 35-40 | 2 | Elect CS | 39 | 4 weeks | BF+Ex | N/U | - |
| 002 | 30-34 | 1 | Em CS | 33 | 2 weeks | Ex Only | 8 weeks | BF+Ex |
| 003 | 30-34 | 1 | NVB | 39 | 2 weeks | BF+Ex | 8 weeks | EBF |
| 004 | 40-44 | 3 | Em CS | 39 | 2 weeks | EBF | 7 weeks | EBF |
| 005 | 25-29 | 3 | NVB | 40 | 3 weeks | EBF | 6 weeks | EBF |
| 006 | 30-34 | 1 | NVB | 38 | 2 weeks | BF+Ex | 8 weeks | EBF |
| 007 | 30-34 | 2 | NVB | 38 | N/U | - | N/U | - |
| 008 | 35-39 | 2 | NVB | 34 | 3 weeks | BF+Ex | 7 weeks | EBF |
| 009 | 30-34 | 2 | NVB | 39 | 2 weeks | BF+Ex | 7 weeks | BF+Ex |
| 010 | 30-34 | 2 | NVB | 39 | 2 weeks | BF+Ex | 8 weeks | BF+Ex |
| 011 | 25-29 | 1 | Em CS | 41 | N/U | EBF | 7 weeks | EBF |
| 012 | 30-34 | 1 | Em CS | 36 | 2 weeks | BF+Ex | N/U | - |
| 013 | 35-39 | 2 | Em CS | 41 | N/U | EBF | 8 weeks | BF+Ex |
| 014 | 30-34 | 1 | Forceps | 39 | 2 weeks | BF+FF | N/U | - |
| 015 | 30-34 | 2 | Em CS | 37 | 2 weeks | EBF | 6 weeks | EBF |
| 016 | 25-29 | 2 | Elect CS | 39 | N/U | EBF | 9 weeks | EBF |
| 017 | 35-39 | 1 | Elect CS | 30 | 6 weeks | BF+Ex | 8 weeks | BF+Ex |
| 018 | 35-39 | 1 | Em CS | 37 | 3 weeks | BF+Ex | 5 weeks | BF+FF |
| 019 | 35-39 | 2 | Em CS | 28 | 13 weeks | BF+Ex | 15 weeks | BF+FF |
| 020 | 40-44 | 1 | Em CS | 37 | 3 weeks | BF+Ex | 6 weeks | BF+Ex |
| 021 | 30-34 | 2 | NVB | 30 | 10 weeks | BF+Ex | 13 weeks | BF+Ex |
| 022 | 30-34 | 1 | NVB | 40 | 3 weeks | EBF | N/U | - |
| 023 | 35-39 | 1 | NVB | 37 | N/U | - | N/U | - |
| 024 | 30-34 | 2+1 | Em CS | 36 | 2 weeks | BF+Ex | N/U | FF^≠^ |
| 025 | 35-39 | 2 | NVB | 36 | 3 weeks | BF+Ex | N/U | BF+FF |
| 026 | 30-34 | 2 | Em CS | 37 | 2 weeks | EBF | N/U | - |
| 027 | 35-39 | 1 | Forceps | 38 | 7 weeks | BF+Ex | N/U | - |
| 028 | 30-34 | 1 | NVB | 40 | N/U | EBF | 5 weeks | EBF |
| 029 | 30-34 | 1 | NVB | 39 | N/U | - | 6 weeks | EBF |
| 030 | 35-39 | 1 | Em CS | 36 | N/U | - | N/U | - |

N/U=not undertaken; Elect CS=Elective Caesarean Section; EM CS=Emergency Caesarean Section; NVB=Normal Vaginal Birth; EBF=exclusive breastfeeding; BF+Ex=breastfeeding and expressing; Ex Only= expressing only; FF=formula feeding; BF+FF=breastfeeding with formula supplement
